# Access Barriers and Variable Response to Semaglutide in Severe Adolescent Obesity: A Case Series

**DOI:** 10.1155/crpe/5562574

**Published:** 2026-06-25

**Authors:** Angela-Tu Nguyen, Jessica L. Binkley

**Affiliations:** ^1^ Department of Pediatrics, Oregon Health & Science University, Portland, Oregon, USA, oregon.gov

**Keywords:** anti-obesity medication, case report, pediatric obesity, semaglutide, treatment access

## Abstract

Pediatric obesity is a worsening public health crisis, affecting 1 out of 5 youth in the United States. Once‐weekly semaglutide has produced clinically meaningful BMI reduction in adolescents in randomized trials. However, trial cohorts and protocols may not reflect real‐world care where patients are more diverse and may face access barriers—a factor that disproportionately affects minoritized groups. In this case report, we describe three adolescents of color with severe obesity treated in a multispecialty obesity clinic with lifestyle counseling and semaglutide. We summarize their clinical course, tolerability, access timelines, and BMI responses. All three reported improved appetite regulation and minimal side effects; however, their trajectories differed as their access and treatment courses varied, which likely affected their BMI outcomes. We will also discuss contextual factors that the general pediatrician should consider when interpreting BMI outcomes, including pubertal stage, gender and body composition, and physical activity. These cases highlight a gap in the literature between efficacy trials and real‐world experience that involves access barriers and developmental factors in a diverse adolescent population.

## 1. Introduction

Pediatric obesity prevalence has increased globally over the past 5 decades, including in the United States where it affects 1 in 5 children [1]. Rates of metabolic comorbidities such as metabolic dysfunction–associated liver disease (MASLD), type 2 diabetes, dyslipidemia, and hypertension are also rising, especially among those with severe obesity [2]. In response, the American Academy of Pediatrics (AAP) published its first clinical practice guideline (CPG) in 2023, recommending intensive health behavior and lifestyle treatment (IHBLT) for children aged 6 years and older and consideration of pharmacotherapy, including glucagon‐like peptide 1 receptor agonists (GLP‐1RAs) such as semaglutide, for those aged 12 years and older [3]. However, access to these treatments is inequitable and may be limited by the availability of multidisciplinary obesity treatment programs or by medication cost [4]. In the STEP TEENS randomized trial, adolescents receiving semaglutide plus lifestyle interventions experienced a mean body mass index (BMI) change of −16.1% after 68 weeks, substantially greater than placebo [5]. Although promising, evidence gaps remain including long‐term outcomes and representation across clinical contexts and pubertal stages. These gaps are important to recognize given that youth from minority racial and ethnic groups are disproportionately affected by obesity and may face inequities in access to obesity treatment [1, 6]. We present the real‐world experiences of three adolescents treated with semaglutide alongside multidisciplinary care in our pediatric obesity clinic by a pediatrician, psychologist, registered dietician, and physical therapist. We highlight treatment decisions, outcomes, and access barriers that may shape BMI trajectories outside of clinical trial environments.

## 2. Case Presentations

Patient 1: A 13‐year‐old male, self‐identified as biracial Black/African American and White, with a BMI of 190% of the 95th percentile (*z*‐score: 4.56), mixed hyperlipidemia, and elevated alanine transaminase (ALT). He was known to be heterozygous for GNAS and PCSK1, genes associated with severe and early‐onset obesity, although the family denied excessive hunger. Upon establishing care, he was weight training 2‐3 times a week. His diet was varied and mostly home‐cooked. He reported snoring and daytime somnolence, but he maintained a regular sleep schedule. His exam was notable only for faint, thin abdominal striae, and his Tanner stage was estimated at 3. We initiated semaglutide, with his family preferring to pay the $1400/month cost rather than delay treatment because of insurance barriers. His medication was titrated to the treatment dose of 2.4 mg weekly per manufacturer’s guidance without adverse effects. He reported reduced hunger overall and earlier satiety with meals. After 5 months at 2.4 mg, the pharmacy hesitated to dispense a refill, seeing insurance had denied coverage (unaware the family was self‐paying), but the patient preferred to discontinue anyway. At discontinuation, his BMI was 174% of the 95th percentile (*z*‐score: 3.88), representing a BMI reduction of 5.7% from semaglutide initiation (4.2% reduction during treatment dosing). At 4 months post‐discontinuation, he regained 17% of his BMI loss, and at 7 months, he had regained it all. Despite this return to baseline, he elected not to restart medication. Throughout this time, he maintained regular clinic appointments every 3‐4 months and worked on packing lunch for school, limiting soda, eating breakfast more regularly, and adding cardiovascular exercise to his routine. He was also referred to sleep medicine to evaluate for obstructive sleep apnea.

Patient 2: A 12‐year‐old male, self‐identified as Black/African American, with a history of mixed hyperlipidemia. He had started semaglutide 5 months prior, prescribed by his primary care provider as a compounded medication for affordability, and had recently reached 2.4 mg weekly. Shortly before semaglutide initiation, his BMI was 170% of the 95th percentile (*z*‐score: 3.82). He played football and basketball and performed strength training 1‐2 times a week. His diet was varied, although his family reported excessive portions prior to semaglutide. After starting semaglutide, they observed decreased dinner portions (“He used to have 3 hamburgers at a meal and now he eats 1”), but he often skipped breakfast and lunch. He occasionally snored, which improved after semaglutide, and he maintained a normal sleep schedule. He managed occasional constipation as a side effect of the medication with a stool softener. His exam was notable for cervical acanthosis nigricans and thin abdominal striae, and he was reportedly at Tanner stage 1. Over the next 8 months, he followed up with our clinic twice and worked on eating three meals a day and using MyPlate for dietary guidance. After eight months on treatment dosing, he was lost to follow‐up, and his final BMI was 141% of the 95th percentile (*z*‐score: 2.72), representing a 13.7% BMI reduction.

Patient 3: A 13‐year‐old female, self‐identified as biracial Black/African American and White, with a BMI of 214% of the 95th percentile (*z*‐score: 5.88), obstructive sleep apnea status post tonsillectomy and adenoidectomy, prediabetes, hypertension, chronic abdominal pain, and posttraumatic stress disorder. She had completed a treatment program for a binge eating disorder years prior. She did not participate in sports or physical education but was active in band and reported more than 10,000 steps/day. Her diet was varied. She ate breakfast and lunch provided by school; about half of her dinners were home‐cooked. Physical exam was notable for cervical acanthosis nigricans and thin abdominal striae, and she was post‐menarcheal. She focused on lifestyle changes for 6 months, including meal planning, closing movement rings on her Apple Watch, limiting eating out, and reducing sugar‐sweetened beverages and processed snacks. When her BMI did not improve, topiramate monotherapy was initiated (off‐label use for obesity) as her insurance company required a failed trial of an oral medication and “Qsymia” (phentermine/topiramate extended‐release) was deemed an inferior choice due to her hypertension. Over eight months, topiramate was titrated to 75 mg daily, but her BMI increased to 233% of the 95th percentile (*z*‐score: 6.53). Topiramate was discontinued, and semaglutide was initiated with Medicaid coverage. The dose was titrated per manufacturer’s guidance to the treatment dose of 1.7 mg weekly. During the titration process, she reported mild nausea with each dose increase, a mild decrease in hunger overall, earlier satiety, and lost 5.2% of her BMI before reaching this dose. After 1 month at the treatment dose, she had regained approximately half of her weight lost. Her last recorded BMI was 223% of the 95th percentile (*z*‐score: 6.02). Throughout this time, she was seen every 3‐4 months for lifestyle counseling.

## 3. Discussion

These patients presented with severe obesity and metabolic comorbidities, received semaglutide, and participated in concurrent lifestyle counseling. None had physical or developmental disabilities. Factors that may have shaped their differing trajectories included sex/gender, pubertal stage, baseline physical activity, lifestyle goals, and access to semaglutide. Comparisons are limited by differences in treatment duration, achieved dose, and total hours of lifestyle counseling received. Laboratory tests were obtained at medication initiation, but endpoints were unavailable. Therefore, laboratory results were omitted for brevity.

Follow‐up visits in our clinic typically involve 1‐2 h of lifestyle counseling; therefore, none received the full 26 h of IHBLT recommended by the AAP through our clinic alone, which may more realistically mimic the intensity of treatment patients might receive outside of a clinical trial. This small series from a single clinic was not designed to draw comparative conclusions, yet it highlights practice factors that can influence obesity care.

BMI is a practical but indirect proxy for adiposity and obesity risk. It does not account for body composition and can under‐ or over‐diagnose obesity in certain groups [7]. Despite its limitations, BMI is also commonly used to assess efficacy in medication trials. Given that GLP‐1RAs are approved for age 12 and up, many patients will be entering or progressing through puberty, when body proportions, body composition, and fat distribution change and sex‐based differences become more pronounced [8]. Additionally, as seen in our cases, patients may engage in varying levels of resistance training, which can affect lean body mass at the start or during weight loss. Together, these factors complicate interpretation of BMI response to GLP‐1RAs and may contribute to variability between clinical trial results and the real world, particularly when most of the trial cohort [5] was female and post‐pubertal.

The path to accessing semaglutide for these patients varied, with barriers like high out‐of‐pocket costs and insurance hurdles. This reflects the common financial barriers to equitable distribution of medications across the U.S. contributing to disparities in obesity care access [9]. To fill the gap, compounded GLP‐1RAs are being increasingly used, leading to safety concerns and the rise of fraudulent medications [10]. Patient 3 had to demonstrate lifestyle changes and fail a trial of a medication known to be inferior in efficacy before her insurance approved semaglutide, and this delay resulted in a rise in her BMI before treatment could begin. These barriers underscore why the AAP no longer recommends a staged approach to treating obesity, and we should advocate for insurance companies to do the same.

We mention race not as a biological determination of health but to provide social context. Racism and related forms of oppression shape access to healthcare as well as physical health factors that contribute to obesity (e.g., chronic stress and lack of access to resources) [3, 6]. Nearly half of U.S. adolescents identify as non‐white, yet youth of color are more likely to experience obesity compared to white teens [11, 12]. Unfortunately, as in the adults, they are less likely to receive or see treatment response to lifestyle treatment, pharmacotherapy, and surgery [6, 13, 14]. They also remain underrepresented in research focusing on obesity treatment, including the clinical trial for semaglutide where only 21% identified as non‐white [5]. We must be mindful of inequities in access to care, including access to GLP‐1RAs, as these access inequities may widen the racial disparities in the obesity epidemic itself [15].

### 3.1. Patient Perspectives

The patients and families were supportive of sharing their experiences for this report and hoped their stories would help providers better understand the lived experience of adolescents receiving medical treatment for obesity. They described treatment as more than taking a medication, requiring patience, persistence, and hard work to make lifestyle changes, attend appointments, and navigate the healthcare system. They hoped their stories will teach providers to recognize the practical and systemic challenges families face when seeking treatment for pediatric obesity.

## 4. Conclusion

We highlight practical considerations for clinicians utilizing GLP‐1RAs in adolescents for obesity:

1. Anticipate access barriers and advocate for timely initiation, treatment continuity, and insurance parity to reduce inequities.
2. Interpret BMI with caution when diagnosing obesity and assessing response to treatment. Pubertal stage, body composition, and baseline and changing physical activity can influence BMI trajectories.
3. Define response beyond weight change, incorporating improved appetite regulation, quality of life, function, cardiometabolic lab markers, and health‐promoting behaviors.

**Nomenclature**
AAP: American Academy of Pediatrics
ALT: Alanine transaminase
BMI: Body mass index
CPG: Clinical practice guideline
GLP‐1RA: Glucagon‐like peptide 1 receptor agonist
IHBLT: Intensive health behavior and lifestyle treatment
MASLD: Metabolic dysfunction–associated liver disease

## Author Contributions

Dr. Angela‐Tu Nguyen conceptualized this case report, collected data, drafted the initial manuscript, and critically reviewed and revised the manuscript.

Dr. Jessica L. Binkley drafted the initial manuscript and critically reviewed and revised the manuscript.

## Funding

No funding was secured for this study.

## Disclosure

All authors approved the final manuscript as submitted and agree to be accountable for all aspects of the work.

## Ethics Statement

This case report was reviewed by the Oregon Health & Science Institutional Review Board and was determined to be exempt from further review. Written informed consent for publication of de‐identified cases was obtained from each adolescent’s legal guardian, with assent from the adolescents.

## Conflicts of Interest

The authors declare no conflicts of interest.

## Data Availability

Data sharing is not applicable to this article as no datasets were generated or analyzed during the current study.
